# Current challenges in the prevention and management of post-thrombotic syndrome—towards improved prevention

**DOI:** 10.1007/s12185-023-03651-6

**Published:** 2023-08-31

**Authors:** Julie Wang, Elise Smeath, Hui Yin Lim, Harshal Nandurkar, Hong Kuan Kok, Prahlad Ho

**Affiliations:** 1 Northern Health, Epping, Melbourne, VIC Australia; 2 Australian Centre for Blood Diseases, Melbourne, VIC Australia; 3 University of Melbourne, Melbourne, VIC Australia; 4 Department of Haematology, Northern Hospital, 185 Cooper St., Epping, Melbourne, 3076 VIC Australia

**Keywords:** Post-thrombotic syndrome, Deep vein thrombosis, Biomarkers

## Abstract

Post-thrombotic syndrome (PTS) is a common and potentially debilitating complication of deep vein thrombosis (DVT), affecting up to 50% of DVT patients. The consequence of this chronic condition includes reduced quality of life, increased use of the healthcare system and decreased productivity. The societal impact of this condition is projected to increase, given our ageing population and increased burden of thrombotic diseases. Despite significant recent advances in our understanding of PTS, many unanswered questions remain. Currently, there are few effective and proven options for established PTS; hence, the emphasis should be on instituting effective prevention to reduce the progression to PTS. Effective anticoagulation lowers the risk of PTS, with direct oral anticoagulants appearing to outperform vitamin-K antagonists. However, the evidence for elastic compression stockings and endovascular thrombolysis or thrombectomy techniques remains unclear. Accurate identification of individuals at high risk of developing PTS may also improve the targeting of preventative interventions. This review will examine the current body of evidence regarding PTS, with a focus on preventative strategies as well as novel biomarkers.

**Supplementary Information:**

The online version contains supplementary material available at 10.1007/s12185-023-03651-6.

## Background

Post-thrombotic syndrome (PTS) is a common condition that afflicts up to 30–50% of individuals following deep vein thrombosis (DVT) [1–5]. It can be diagnosed from 3 to 6 months after an episode of DVT [6–8]. Symptoms and signs of PTS overlap with primary venous insufficiency and include leg pain, heaviness, fatigue, swelling, skin discolouration, lipodermatosclerosis and venous ulcers in severe cases. Symptoms are typically exacerbated by standing or walking and can range from mild to severe in up to 15% [9].

PTS is a major cause of reduced quality of life (QOL) [10–13], comparable to that caused by diabetes or chronic lung disease [13]. The presence of PTS is the main determinant of general and venous disease-specific QOL at 2 years after DVT diagnosis [13] and its impact appears to be similar for proximal and distal DVT [10]. PTS also adds to the health economic costs of DVT [14]. A Canadian prospective study of acute DVT patients [15] found that PTS was associated with a 35–45% increase in costs to the patient and healthcare system, including 62% attributable to non-medical costs such as loss of productivity [15]. Despite this, PTS is under recognised by many physicians, further compounding the QOL impact for these patients. Given that the global incidence of DVT is 0.5–1.5/1000 population [16], the total societal impact of PTS is substantial and one that could potentially be mitigated with effective preventative strategies.

The purpose of this review is to appraise the current body of knowledge about PTS, with an emphasis on preventative strategies including novel biomarkers. The treatment modalities for PTS remain understudied and only modestly effective. As a result, more emphasis should be placed on preventing the development of PTS.

**Fig. 1:**
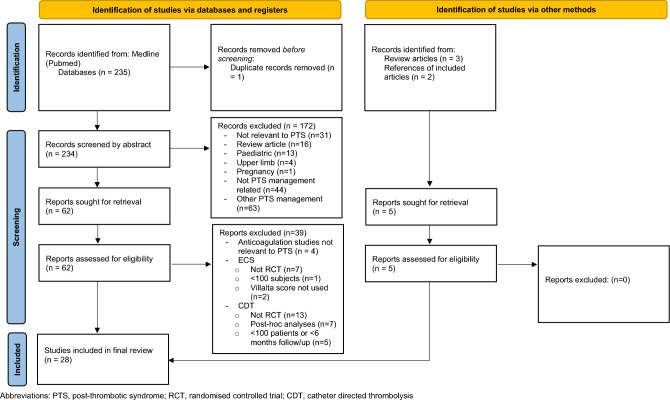
PRISMA diagram outlining the research of search strategy and criteria used to obtain the final included studies. PTS, post-thrombotic syndrome; RCT, randomised controlled trial; CDT, catheter-directed thrombolysis

A systematic review of the literature was conducted to compile current evidence for the section on preventative treatments. The Medline (PubMed) database was searched on 5 July 2022, using the search strategy set out in the supplementary materials. The resulting search yielded 235 studies. Criteria used in identifying, screening and excluding studies are outlined in the PRISMA diagram (Fig. 1). The resulting 28 studies were compiled into three sections with anticoagulation in Table 2, elastic compression stockings in Table 3 and catheter-based early thrombus removal in Table 4.

### Pathophysiology

**Fig. 2:**
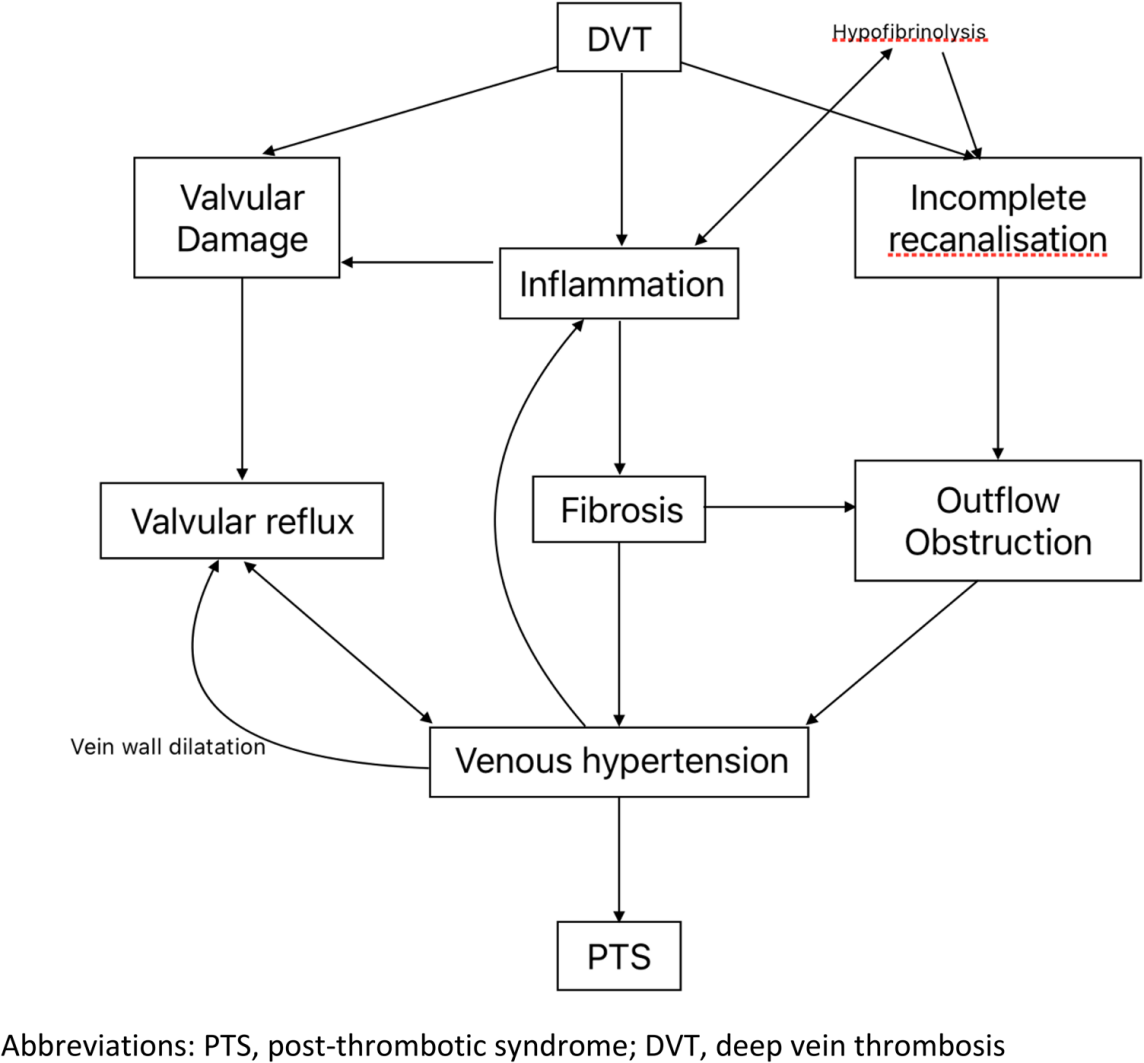
Proposed schematic diagram of the progression to PTS after DVT. PTS, post-thrombotic syndrome; DVT, deep vein thrombosis

The pathophysiology of PTS is incompletely understood, but is believed to result from venous hypertension caused by venous valvular damage and venous outflow obstruction from residual thrombosis and vessel wall fibrosis [17, 18] (Fig. 2). Venous hypertension and alterations of blood flow contributes to a chronic state of inflammation, by activating the endothelium and upregulating the expression of leucocyte adhesion molecules, causing release of pro-inflammatory cytokines and disrupting the glycocalyx [19–22]. This pro-inflammatory response and the consequences of the interaction of the thrombus with the vessel wall are likely integral to the pathogenesis of PTS [23]. However, due to the difficulties of collecting human deep vein tissues, our understanding of the molecular basis of PTS is mostly based on animal models. Recent human radiological studies showing thicker venous walls in PTS patients compared to acute DVT and normal controls support a role for venous vascular remodelling in the pathophysiology of PTS [24, 25].

Impaired fibrinolysis has been implicated in the development of PTS as residual thrombus is a known risk factor for PTS. However, measurements of individual components of the fibrinolytic system in PTS patients have shown conflicting results [26]. Besides its primary action to break down fibrin, plasmin(ogen) facilitates wound repair via a variety of plasminogen receptors, including those found on neutrophils and macrophages, which are the key cells involved in deep vein thrombosis resolution [27, 28]. Critical to this regenerative capacity is the ability of plasmin to directly activate metalloproteinases (MMP) from proMMPs, which act to degrade extracellular matrix and basement membrane components [29]. MMP-2 and MMP-9 have been shown to be increased following DVT [30, 31]. MMP-9 deleted mice showed increased vessel wall stiffness during thrombus resolution [32, 33]. Elevated PAI-1 leads to reduced MMP-2 and MMP-9 activity and vein wall fibrosis [34]. Venous thrombosis also causes the release of mediators including TGFb, IL-13 and MCP-1 which further promotes fibrosis [31, 35]. The fibrinolytic system could therefore be attractive targets for prevention of PTS. Despite a large number of candidate compounds, none have entered human trials to date [36]. The complex interplay of the thrombotic, fibrinolytic and inflammatory systems, together with our incomplete understanding of PTS pathogenesis, adds to the difficulty in finding effective therapeutic targets in PTS.

### Risk factors for PTS

The recognised risk factors for development of PTS are listed in Table 1. The most important of these include proximal location of DVT (especially femoral and iliac veins), recurrent DVT history and obesity [3–5, 37–42]. Pre-existing chronic venous disease [37] and the presence of venous insufficiency signs in the contralateral leg [3] are risk factors for development of PTS and raise the possibility that primary venous insufficiency and PTS may share mechanistic pathways. An association with female sex is shown in some studies [2, 37, 41], but not others [3–5, 38, 39]. Thrombophilia, however, is not a proven risk factor [37, 43].

**Table 1:** Risk factors for development of PTS

Risk factors at baseline	Risk factors during follow-up
Proximal DVT (especially iliofemoral)	Subtherapeutic INR [41]
Recurrent DVT history	Ipsilateral recurrent DVT [123]
Obesity [38]	Residual thrombus [3, 102, 124]
Pre-existing venous insufficiency	Venous reflux [102]
Higher severity of symptoms at diagnosis [39, 42]	
Older age [125]	

PTS, post-thrombotic syndrome; DVT, deep vein thrombosis; INR, international normalised ratio

### Diagnosis

The diagnosis of PTS is predominantly based on clinical symptoms, and one of the challenges is the considerable overlap of symptoms between PTS, recurrent DVT and primary venous insufficiency [44]. To help standardise clinical PTS diagnosis, various diagnostic tools have been formulated, each with its strengths and drawbacks [45, 46] (Supplemental Tables S1-3). The Villalta score is currently the most widely used score in both clinical practice and research and has been found to have high inter-observer reliability [47]. It is endorsed by the International Society of Thrombosis and Haemostasis (ISTH) and American Heart Association (AHA) [48, 49]. However, there remains no gold standard adjunctive biomarker or diagnostic test for PTS which makes both the recognition and diagnosis of PTS subjective to symptom reporting and clinician judgement. Additionally, given that many DVT patients have concurrent chronic venous insufficiency [3], PTS may also represent the natural progression or acceleration of an underlying and pre-existing chronic venous disease process.

PTS symptoms can mimic those of acute DVT, and in patients with recurrent symptoms in the ipsilateral leg, compression duplex ultrasonography (CUS) may be unable to distinguish acute from chronic thrombus [50] and previous imaging may not always be available for comparison, particularly if follow-up care is fragmented. Magnetic resonance (MR) venography is more accurate than CUS, particularly in the iliac veins, and also permits more accurate detection of venous wall scaring and venous inflow assessment [51, 52]. Recently, magnetic resonance direct thrombus imaging (MRDTI) has been shown to be highly sensitive and specific in the diagnosis of recurrent DVT. This method is based on the detection of high signal in T1-weighted MRI from methaemoglobin within blood clots, which disappears completely after 6 months. In a prospective study of 305 patients [53] with suspected recurrent ipsilateral DVT, inconclusive diagnoses were reduced from 30% to < 1% using MRDTI, with only 2 patients (1.7%) developing VTE within 30 days. MRDTI does not require contrast, is quick to perform and has excellent reproducibility between observers and across centres; it has the potential to change practice in this challenging patient population. Other imaging findings that can support a PTS diagnosis include luminal stenosis or narrowing, fibrotic bands, venous reflux and the presence of collateral veins [50]. CUS, CT venography and/or MR venography each have respective roles, but is substantially influenced by experience and familiarity between centres.

## Current preventative strategies

Prevention remains the mainstay of PTS management and involves a combination of effective anticoagulation, use of elastic compression stocking in certain scenarios as well as early identification of high-risk VTE which may benefit from early catheter-based thrombolysis or thrombectomy.

### Anticoagulation

Effective anticoagulation is one of the most effective strategies to prevent PTS, through early thrombus resolution by preventing thrombus propagation, and therefore reducing valve damage and residual vein obstruction, two of the major causes of PTS [54]. However, the duration of anticoagulation to treat DVT is not associated with improved clinical outcome, which was demonstrated in the ExACT randomised controlled trial (RCT) [55]. However, the time in the therapeutic range is critical, with subtherapeutic anticoagulation with vitamin K antagonists (VKAs) shown to increase the risk of PTS in several studies. [3, 41, 56]. This finding raises important questions about the type of anticoagulation used, particularly in the era of direct oral anticoagulants (DOAC), where all but one study shows DOACs to be associated with reduced likelihood of PTS (Table 2), likely in part due to their stable anticoagulation effect. The study that showed no difference [57] was a Danish registry study that relied on the McDougall criteria, based on symptoms and signs in medical records which may not be generalisable to clinical practice. Similarly, studies suggests that LMWH compared to VKAs likely reduce the risk of PTS and improves vein re-canalisation [58–61], of which the benefit conferred by LMWH may be twofold—more stable anticoagulation effect and possible anti-inflammatory effects [62]. Taken together, the body of knowledge to date highlights that effective anticoagulation is pivotal to reduce the occurrence of PTS.

**Table 2:** Studies examining anticoagulation and risk of developing PTS

Reference ID and first author	Type of study	Number of participants	Intervention tested	Length of follow-up	Findings
*Studies comparing DOACs vs VKA*					
Soares 2019 [126]	Prospective randomised cohort study	*N* = 84 1^st^ proximal DVT - Rivaroxaban group: 46 - Warfarin group: 38	Rivaroxaban versus warfarin for 6 months - All patients wore 30-40 mmHg ECS	12 months	PTS (Villalta) at 12 months was significantly higher in warfarin group (28.9%) compared to rivaroxaban group (8.7%) OR 4.278, * p* < 0.001 Venous re-canalisation rate at 12 months was 76.1% in rivaroxaban group compared to 13.2% in warfarin group (* p* < 0.001)
Prandoni 2020 [127]	Multi-centre prospective cohort study	*N* = 1345 -Prospective cohort (DOACs): 309 -Historical cohort (vitamin K antagonists):1036	DOACs versus Vitamin K antagonists (historical cohort) - compression therapy at discretion of clinician	Up to 3 years	PTS (Villalta) developed in 28.2% treated with DOACs vs 42.8% treated with VKAs (adjusted OR 0.46, 95%CI 0.33–0.63)
Jeraj 2017 [128]	Observational cohort study	*N* = 100 consecutive proximal DVTs - Rivaroxaban group: 61 - Warfarin: 39	Rivaroxaban versus warfarin for mean 6 months All patients prescribed compression therapy	36 months	Univariate odds ratio 2.9 for development of PTS (Villalta) in warfarin group (95%CI 1.2–6.8, * p* = 0.014) After adjusting for variables (age, gender, recurrent DVT, time from symptom to diagnosis and then to check-up) patients on warfarin were more likely to develop PTS (OR 3.5 95% CI 1.1–11.0, * p* = 0.035)
Coleman 2018 [129]	Retrospective database study	*N* = 36,957 from US health insurance data—ICD-9 code for DVT or PE - Rivaroxaban group: 10,463 - Warfarin group: 26,494	Rivaroxaban versus warfarin	Mean follow-up of 16 months	PTS defined by MacDougall algorithm, 3 months after DVT diagnosis Risk of PTS for rivaroxaban compared to warfarin was lower, HR 0.77, 95%CI 0.70–0.84, * p* < 0.0001
Søgaard 2018 [57]	Retrospective registry study	*N* = 19,957 first inpatient or outpatient diagnosis of venous thromboembolism from Danish Patient Register, based on ICD-10 codes	Warfarin versus rivaroxaban	3 years	PTS defined by MacDougall algorithm, 3 months after DVT diagnosis No difference in rates of PTS for warfarin or rivaroxaban groups. PTS occurred in 0.53 per 100 person-years for rivaroxaban vs 0.55 per 100 person-years for warfarin, HR = 0.88 (95% CI, 0.66–1.17)
Utne 2018 [130]	Cross-sectional study	*N* = 309 1^st^ DVT after 90 days anticoagulation - Rivaroxaban: 161 - Warfarin: 148	Warfarin versus rivaroxaban	24 months after DVT diagnosis	PTS defined by Patient Reported Villalta Scale Adjusted OR for PTS with rivaroxaban = 0.5 (95% CI 0.3–0.8, * p* = 0.01)
Ferreira 2020 [131]	Retrospective cross-sectional multi-centre cohort study	*N* = 129 proximal DVT after 3 months anticoagulation - Rivaroxaban: 71 - Warfarin: 58	rivaroxaban versus enoxaparin/warfarin	Median follow-up of 15 months rivaroxaban vs warfarin 61 months	PTS (Villalta) developed in rivaroxaban group 50.7% vs warfarin 69%, * p* = 0.018 Risk of PTS in rivaroxaban group OR 0.29, 95% CI 0.10–0.80, * p* = 0.018 after adjusting for age, gender and BMI Lower rate of RVT in rivaroxaban group (24.4% versus 64.6%), OR 0.14 (95% CI 0.1–1.0, * p* = 0.051)
Cheung 2016 [132]	Post hoc analysis EINSTEIN study	*N* = 336 proximal DVT - Rivaroxaban: 162 - VKA: 174	Rivaroxaban versus VKA	60 months	Cumulative incidence of PTS (Villalta) was 29% in rivaroxaban and 40% in VKA group (adjusted HR 0.76, 95%CI 0.51–1.13, * p* = 0.18) ECS use was 69% in rivaroxaban and 80% in VKA group
*Studies comparing LMWH vs VKA*					
Hull 2009 [58]	Multi-centre, randomised controlled trial	*N* = 480 proximal DVT - tinzaparin:240 - tinzaparin and warfarin:240	Tinzaparin once daily for 12 weeks vs tinzaparin for > 5 days plus oral warfarin for 12 weeks	PTS assessed at 12 weeks	PTS symptoms elicited from patient by questionnaire with 8 items Overall OR for PTS symptoms in tinzaparin group was 0.77 (* p* = 0.001)
Gonzalez-Farjardo 2008 [59]	Prospective randomised controlled trial	*N* = 100 1^st^ DVT - enoxaparin prophylactic dose: 56 - VKA: 44	Enoxaparin (40 mg daily) versus VKA for 3 months 40 mmHg ECS all patients for 2 years	PTS assessed after 5 years	Lower incidence of PTS (Villalta) in enoxaparin group vs coumarin although this was not statistically significant A significant inverse correlation between amount of thrombus regression at 3 months and incidence at 5 years of PTS (* p* = 0.007)
Daskalopoulos 2005 [60]	Prospective randomised controlled trial	*N* = 102 proximal DVT - tinzaparin group: 50 - VKA group: 52	Tinzaparin (therapeutic dose) vs VKA for 6 months	12 months	More re-canalisation detected by Doppler ultrasound in tinzaparin group at 3-, 6- and 12-month follow-up Did not measure for PTS symptoms
Romera 2009 [61]	Prospective randomised controlled trial	*N* = 241 1^st^ proximal DVT - LMWH group: 119 - VKA: 122	Tinzaparin (therapeutic dose) vs VKA for 6 months	12 months	Total re-canalisation of the vein was more frequent in LMWH group than VKA group at 6 and 12 months (* p* < 0.001) No measurement of PTS symptoms
*Other studies*					
Bradbury 2020 [55]	Prospective multi-centre randomised controlled trial	*N* = 273 1st unprovoked proximal DVT or PE after 3 months anticoagulation (VKA) -Limited anticoagulation: 134 -Extended anticoagulation (incl * n* = 2 rivaroxaban): 139	limited duration (stopped after 3 months anticoagulation) vs extended duration (24 months) anticoagulation	24 months	Extended anticoagulation: reduction in recurrent VTE (2.75 vs 13.54 events per 100 patient years, adjusted HR 0.20 (95% CI, 0.09–0.46, * p* < 0.001) No difference in outcomes of PTS and quality of life
Van Dongen 2005 [41]	Observational cohort study	*N* = 244 with proximal DVT	Anticoagulation with VKAs for 3 months 30-40 mmHg ECS all patients for 2 years	Median follow-up of 4.9 years	PTS defined by Villalta criteria - Patients who spent > 50% of time with INR < 2.0 associated with increased risk of PTS (OR = 2.71, 95% CI 1.44–5.10)
Chitsike 2012 [56]	Multi-centre prospective cohort study	*N* = 349 1^st^ unprovoked proximal DVT and had received warfarin for 5–7 months	LMWH for at least 5 days followed by warfarin 5–7 months	N/A	In the first three months, adjusted OR for PTS development if INR < 2 for more than 20% of the time was 1.84 (95% CI 1.13–3.01) For the entire period of anticoagulation, adjusted OR for PTS development if INR < 2 for more than 20% of the time was 1.88 (95%CI 1.15–3.07)

RCT, randomised controlled trial; PTS, post-thrombotic syndrome; DVT, deep vein thrombosis; VKA, vitamin K-antagonist; INR, international normalised ratio; DOAC, direct oral anticoagulant; PE, pulmonary embolus; ECS, elastic compression stocking; LMWH, low molecular weight heparin; HR, hazard ratio; OR, odds ratio;

### Elastic compression stockings (ECS)

The mechanism by which ECS exert their beneficial effects are not entirely known. Some of the proposed mechanisms include reduction in the venous hypertension, increased venous flow velocity, reduction in venous reflux and blood volume in legs due to the reduction in the vein diameter of major veins and improving lymphatic drainage due to increase in tissue pressure [63, 64].

The evidence for ECS is mixed with several randomised controlled studies (RCT) comparing ECS to no stockings demonstrating around 50% reduction in PTS incidence (see Table 3). However, the largest placebo-stocking controlled trial of 804 patients (‘SOX’ study) [65] failed to show any interventional effect. In this study, PTS developed in 14.2% of the active ECS group vs 12.7% in the placebo group (HR 1.13, 95% CI 0.73–1.76, *p* = 0.58). However, one reason for the lack of efficacy could be due to the substantially lower compliance rate in this study compared to other RCTs. Barriers to optimal ECS usage may include discomfort, cost and difficulty putting on the stockings. Studies have examined the effect of modifications to the standard ECS regimen to improve compliance and found non-inferior results with reduced compression strength stockings (25 mmHg instead of 35 mmHg) [66], reduced ECS duration to 12 months [67] and an individualised tailored regimen based on Villalta score [68]. These strategies may be adopted in clinical practice to improve compliance with ECS. Nonetheless, the results of the SOX study have proved influential and have resulted in recent VTE guidelines recommending ECS for reduction of symptoms rather than direct prevention of PTS [69, 70].

**Table 3:** Randomised controlled trials of elastic compression stockings for the prevention of PTS

Reference ID and first author	Type of study	Participants	Intervention tested	Length of follow-up	Findings
Prandoni 2012 [6]	Open label RCT	*N* = 267 1st proximal DVT -Thigh length group: 135 -Below knee group: 132 VKA – INR > 70% in range	Thigh length versus below knee compression elastic stockings 30–40 mmHg at hospital discharge, for 2 years	36 months	- PTS (Villalta) developed in 32.6% of thigh length group and 35.6% of below knee group (adjusted HR 0.93, 95% CI 0.62–1.41) - Side effects (itch, erythema, allergy): thigh length 40.7% vs 27.3% below knee (* p* = 0.017) - Self-reported compliance 66.7% in thigh length vs 82.6% in below knee group (* p* = 0.003)
Kahn 2014 [65] ‘SOX’	Multi-centre RCT	*N* = 804 1^st^ proximal DVT - Active ECS group: 409 - Placebo ECS group: 394 VKA – no monitoring INR Previous venous insufficiency included	Active (30-40 mmHg) versus placebo (< 5 mmHg) ECS within 2 weeks DVT diagnosis, for 2 years	24 months	- PTS (Ginsberg) developed in 14.2% of active ECS group vs 12.7% in placebo ECS group (HR 1.13, 95% CI 0.73–1.76, * p* = 0.58) - Active ECS had no effect on PTS incidence by Villalta criteria, venous ulcers, recurrent VTE, venous valvular reflux or quality of life - Self-reported compliance with stockings 55.6% for ≥ 3 days per week by 24 months
Prandoni 2004 [7]	RCT	*N* = 180 proximal DVT -Below knee ECS: 90 -No ECS: 90 VKA – INR > 70% in range	Below knee ECS (30-40 mmHg) vs no ECS at hospital discharge, for 2 years	Up to 5 years	- PTS (Villalta) developed in 49.1% of controls vs 24.5% in ECS group (adjusted HR 0.49, 95%CI 0.29–0.84, * p* = 0.011) - HR for PTS in ECS group compared to control 0.49 (95% CI 0.29–0.84, * p* = 0.011) - Self-reported compliance 93% in the intervention group
Yang 2022 [133]	RCT	*N* = 232 proximal DVT -Below knee ECS: 113 -No ECS: 119	Below knee ECS (30–40 mmHg) vs no ECS, within 28 days DVT diagnosis	24 months	- PTS (Villalta) developed in 42% of the ECS group vs 57.8% in the control group at 24 months (Risk ratio 0.73, 95% CI 0.55–0.96, * p* = 0.024) - Self-reported compliance was at least 6 days/week in 75.2%
Cate-Hoek 2018 [68] ‘IDEAL-DVT’	Multi-centre, non-inferiority RCT	*N* = 865 proximal DVT -Individualised duration of ECS: 437 -standard duration ECS: 428	Individualised duration of ECS (30-40 mmHg) (discontinued if Villalta ≤ 4 at 3 and 6 months) vs 24 months of ECS, within 24 h DVT diagnosis	24 months	- PTS (Villalta) occurred in 28.9% of individualised duration vs 27.8% standard duration (OR for difference 1.06, 95% CI 0.78–1.44) Absolute difference 1.1% (95% CI 5.2–7.3%) meeting the non-inferiority margin - Self-reported compliance with daily ECS use by 24 months was 77% in intervention group and 79% in standard therapy group
Mol 2016 [67] ‘OCTAVIA’	Multi-centre, non-inferiority RCT	*N* = 518 proximal DVT after 12 months of using ECS (34-46 mmHg), without already developed PTS - Stop ECS at 12 months: 256 - Continue ECS for total 24 months): 262	ECS for 12 months vs 24 months	12 months from randomisation	- Stop ECS at 12 months group developed PTS in 19.9% vs Continue ECS group developed PTS in 13.0% - Did not meet non-inferiority endpoint—adjusted HR of Stop ECS group 1.6 (1.02–2.5) - Self-reported compliance was 85% using ECS 6–7 days a week
Brandjes 1997 [8]	RCT	*N* = 194 1st proximal DVT - ECS group: 96 - no ECS group: 98	ECS (21-40 mmHg) vs no ECS, applied within 2–3 weeks of DVT diagnosis, for 2 years	At least 60 months	- PTS was diagnosed with a custom score and divided into mild–moderate PTS or severe PTS - Mild–moderate PTS developed in 20% of ECS group vs 47% of no ECS group (* p* < 0.001) - Severe PTS developed in 11% of ECS group vs 23% of no ECS group (* p* < 0.001) - Daily compliance of stockings was observed in 76% of patients
Galanaud 2022 [66] ‘CELESTE’	Double-blinded RCT	*N* = 350 1st proximal DVT, at ≤ 8 days of diagnosis -25 mmHg ECS group: 175 -35 mmHg ECS group: 175	25 mmHg ECS vs 35 mm Hg ECS for 2 years Patients could choose thigh length or knee length	24 months	- PTS (Villalta) developed in 31% in 25 mmHg group vs 33% of 35 mmHg group. Absolute difference -2.3% (90%CI -12.1 to 7.4, * p* = 0.0062 for non-inferiority) - PTS developed in 26% of reasonably adherent vs 40% of non-reasonably adherent (* p* = 0.0018) - Self-reported compliance 56% wearing ECS > 80% of the time

RCT, randomised controlled trial; PTS, post-thrombotic syndrome; DVT, deep vein thrombosis; VKA, vitamin K-antagonist; INR, international normalised ratio; ECS, elastic compression stocking; LMWH, low molecular weight heparin; HR, hazard ratio; OR, odds ratio;

### Catheter-based early thrombus removal

Early thrombus removal can rapidly improve venous circulation in symptomatic iliofemoral DVT. It may also prevent PTS development in this high-risk group by removing thrombus at an early stage when thrombolysis or thrombectomy techniques are more likely to be effective. Endovascular techniques include catheter-directed thrombolysis (CDT) where thrombolytics are administered through a multi-sidehole infusion catheter placed across the thrombosed venous segment and pharmacomechanical catheter-directed thrombolysis (PCDT) in which an endovascular device macerates or extracts the thrombus in conjunction with thrombolysis, thus reducing the dose and duration of thrombolysis. To date, however, the data for the use of these methods remain relatively mixed, with important questions still to be clarified.

Table 4 summarises the results of three high-quality randomised controlled trials of CDT/PCDT. The CaVenT study [71] randomised 209 patients with iliofemoral DVT to CDT or standard therapy with anticoagulation alone within 21 days of symptom onset. At two-year follow-up, a minor interventional effect was found (PTS rates 41.1% vs 55.6%, *p* = 0.047). The final 5-year CaVenT [72] results, however, demonstrated a more marked difference in the intervention group (PTS rates 43% vs 71%, *p* = 0.001). Major bleeding in CaVenT occurred in 2.7% of the CDT group vs none in the control group. The CAVA study [73] randomised 152 patients to receive ultrasound accelerated catheter-directed thrombolysis (UACDT) or standard therapy in iliofemoral DVT within 21 days of symptom onset. At 1 year, PTS rates were no different for both groups (OR 0.75, 95% CI 0.38–1.50, *p* = 0.42), although the final follow-up (median 39 months) demonstrated lower PTS rates in the interventional group (46.8% vs 69%, OR 0.40 95%CI 0.19–0.84, *p* = 0.01) [74]. Major bleeding occurred in 5% of the CDT group vs none in the control group.

**Table 4:** Large RCTs of catheter-based early thrombus removal for the prevention of PTS

Reference ID and first author	Type of study	Number of participants	Intervention tested	Length of follow-up	Findings
Vedantham 2017 [75] ‘ATTRACT study’	Multi-centre RCT	*N* = 692 DVT involving FV, CFV or iliac veins within 14 days symptom onset; established PTS excluded; all pts used ECS - thrombolysis group: 337 - no thrombolysis group: 355	Pharmacomechanical thrombolysis (AngioJet or Trellis) +—stenting vs control	24 months	- No difference in PTS (Villalta + venous ulcer) rates at 24 months: 47% in thrombolysis group vs 48% in control group (RR 0.96, 95%CI 0.82–1.11, * p* = 0.56) - Lower rates moderate-to-severe PTS in PCDT group (RR 0.73 95%CI 0.54–0.98, * p* = 0.04) - Mean Villalta score and VCSS score higher at all time points in control - Early major bleeding 1.7% in intervention vs 0.3% in control (RR 6.18, 95%CI 0.78–49.2, * p* = 0.049) - No difference in recurrent DVT - 14.6% of controls vs 8.3% in intervention missed all 4 PTS assessments and 30.7% vs 27.8% missed 1–3 PTS assessments:
Notten 2020 [73] ‘CAVA study’	Multi-centre RCT	*N* = 152 1^st^ iliofemoral DVT within 14 days symptoms; pre-existent venous insufficient signs excluded; all pts used ECS - UACDT group * n* = 77 - Standard group * n* = 75	UACDT (Ekos Endowave) (± stenting) vs Standard treatment	1 year	- No difference in PTS rates: 29% in UACDT patients 35% in Standard group OR:0.75 95% CI 0.38–1.50, * p* = 0.42 - Major bleeding in 4 UACDT patients (5%) and one patient who also had associated neuropraxia - No difference in recurrent DVT rates (non-stent) - 13% suffered in-stent thrombosis in UACDT group - Major bleeding occurred in 5% thrombolysis vs 0 in Standard group
Notten 2020 [74] ‘CAVA final analysis’	Multi-centre RCT	*N* = 120 in final follow-up - UACDT group * n* = 62 - Standard group * n* = 58	UACDT (Ekos Endowave) (± stenting) vs Standard treatment	Median follow-up of 39 months (IQR 23.3–63.8)	- No difference in PTS rates: PTS (Villalta) in 19 (30.6%) UACDT vs 26 (44.8%) in standard group. OR 0.54, 95% CI 0.26–1.15 * p* = 0.11 - When using Villalta + venous ulcer presence, PTS was significantly reduced 29 (46.8%) vs 40 (69%) OR 0.40 95% CI 0.19–0.84, * p* = 0.01 - No major bleeding occurred after the 12-month follow-up
Enden 2012 [71] ‘CaVenT study’	Multi-centre RCT	*N* = 189 DVT above mid-thigh within 21 days symptoms - standard treatment: ECS and anticoagulation: 99 - standard treatment + CDT: 90	- CDT vs standard treatment - all VKA for min 6 months - all class II stockings	24 months	- At 6 months: no difference in PTS rates (30.3% v 32.2%, * p* = 0.77) - At 24 months: lower PTS in CDT group (41.1% vs 55.6%, * p* = 0.047) - 20 bleeding complications in CDT group (3 major) vs none in standard group
Haig 2016 [72] (CaVenT 5-year follow-up)	Multi-centre RCT	*N* = 176 at final follow-up; 1^st^ high proximal DVT (higher than proximal half of femoral vein) within 21 days of symptoms - standard treatment: ECS and anticoagulation: 89 - standard treatment + CDT: 87	- CDT vs standard treatment - all VKA for min 6 months - all class II stockings	5 years	- 43% developed PTS in CDT group versus 71%in standard treatment (95% CI 61–79%, * p* < 0.0001). Absolute risk reduction 28% (95% CI 14–42%). Number needed to treat 4 - Scores on quality of life showed no difference between groups - Recurrent VTE 13(15%) in CDT and 21 (24%) in Standard groups

PTS, post-thrombotic syndrome; DVT, deep vein thrombosis; VKA, vitamin K-antagonist; ECS, elastic compression stocking; HR, hazard ratio; OR, odds ratio; RCT, randomised controlled trial; FV, femoral vein; CFV, common femoral vein; RR, relative risk; UACDT, ultrasound-assisted catheter-directed thrombolysis; IQR, inter-quartile range; CDT, catheter-directed thrombolysis;

The ATTRACT study [75] randomised 692 patients including both iliofemoral and femoropopliteal DVTs, to various PCDT techniques (including AngioJet or Trellis pharmacomechanical thrombectomy) against standard therapy. No differences in PTS rates were found at 24 months; however, there was a significant but modest reduction in moderate-to-severe PTS as a composite secondary post hoc outcome in the PCDT subgroup with acute iliofemoral DVT (see Table 4). Major bleeding occurred in 1.7% of PCDT vs 0.3% in the control group (RR 6.18, 95%CI 0.78–49.2, *p* = 0.049). Several potential causes for the weak effect sizes have been identified in post hoc analyses. PCDT may not be effective for femoropopliteal DVTs (43% of study patients) [76] and is less likely to benefit those over the age of 65 [75]. In addition, PCDT did not significantly reduce incidence of valvular reflux, despite reducing residual thrombus volume [77]. The optimal timing of intervention is also unclear, but it is generally accepted that early intervention derives the greatest benefit. Post hoc analysis of ATTRACT found that intervention within 4–8 days from symptom onset was associated with the greatest benefits in QOL and Villalta scores. Interestingly, no significant improvement was seen in those who received intervention at < 4 or > 8 days [78]. Long-term data from ATTRACT have not been released, but if made available, they may provide additional insights.

The current evidence for endovascular intervention remains strongest in patients with acute, proximal iliofemoral DVT where there are potential gains in reducing the incidence and severity of PTS. However, the choice of CDT or PCDT remains uncertain and ATTRACT has not provided a satisfactory answer in this regard, particularly considering the high consumable cost associated with mechanical thrombectomy and the higher risk of clinically major bleeding compared to anticoagulation alone. Nevertheless, this is an important and active area of research and has the potential to greatly improve outcomes in a high-risk group. There is a need for more tailored randomised clinical trials that address the findings and deficiencies of previous studies. This includes defining the best group to target to maximise interventional benefit, as well as designing studies to address the optimal timing of intervention and the most effective type of catheter thrombus removal technique as well as cost–benefit analysis of newer generation devices over CDT alone.

### Other pharmacotherapy as preventative agents

Several drugs with anti-inflammatory effects are candidates for prevention of post-thrombotic syndrome (PTS), but evidence to date is limited. Statins have been found to reduce venous thromboembolism (VTE) rates in a large RCT (HR 0.61, 95%CI 0.37–0.86, *p* = 0.007) [79], but their impact on PTS prevention is uncertain. The recent SAVER pilot RCT [80] did not show any difference in PTS, including in the DVT subgroup although a larger trial is planned (ClinicalTrials.gov NCT04319627). Sulodexide, an orally bio-available glycosaminoglycan mixture, possesses antithrombotic, anti-inflammatory and endothelial protective effects without increasing the risk of bleeding [81]. Although widely used in Europe, it does not have approval from the US Food and Drug Administration (FDA) or Australian Therapeutic Goods Administration (TGA) [82]. The SURVET RCT [83] found that sulodexide after the initial anticoagulation phase of DVT reduced recurrence of VTE compared to placebo (adjusted HR 0.45, 95%CI 0.24–0.84; *p* = 0.01) without increasing bleeding. Its effectiveness in preventing PTS has not been well studied, with only an Italian registry study [84] showing reduced PTS rates at 5 years in the sulodexide group (12.17% vs 18.23% *p* < 0.05). Evidence is lacking on the efficacy of venoactive agents such as diosmin and rutosides in preventing PTS, despite their use to relieve symptoms of chronic venous insufficiency [85]. A small RCT investigating Diosmin 600 [86] found significantly lower PTS in the intervention group after 12 months following iliofemoral DVT. A larger RCT of micronised purified flavonoid fraction (MPFF) is in the planning phase [87]. The results of these and other randomised trials of promising compounds are anticipated with interest.

## Treatment of established PTS

The treatment of established PTS is challenging and there have only been a few studies that have specifically explored treatment modalities in this setting. Current strategies are mostly extrapolated from chronic venous disease. This section discusses current limited evidence for the treatment of established PTS.

### Compression therapy

While there is evidence to support the use of ECS in chronic venous disease to relieve symptoms and improve QOL [88], there is limited evidence for the use of ECS in established PTS. In an RCT including 35 patients with established PTS, ECS was not effective in reducing PTS symptoms compared to placebo [89], and a 2019 Cochrane review [90] of ECS also determined that there was very low-certainty evidence to support ECS or pneumatic devices in this setting, and that further studies were required.

### Exercise

Exercise may improve venous blood return by improving calf and thigh pump function. A small RCT of 43 PTS patients [91] found no differences in Villalta scores after a 6-month exercise programme, but significant improvements were seen in VEINES-QOL, leg strength and quadriceps flexibility. Evidence from chronic venous disease [92, 93] suggests that exercise can improve symptoms, QOL and muscle function. Although the evidence is limited, exercise programmes are unlikely to be harmful, and the American Heart Association guidelines recommend a 6-month structured exercise programme in PTS patients who can tolerate it [49].

### Pharmacotherapy

There is currently no proven pharmacological therapy for established PTS. Anticoagulation can prevent recurrent DVT, lowering the risk of further vasculature damage, but it has no direct effect on PTS. Venoactive agents are widely used for chronic venous disease in many parts of the world, but the level of evidence in this setting is low [85]. Few studies have specifically examined these compounds in PTS, and a 2018 Cochrane review of rutosides in PTS found no benefit [94]. Sulodexide can reduce symptoms of chronic venous disease [95], and several RCTs found that it improved venous ulcer healing rates [96]. However, its use is restricted due to the lack of FDA and TGA approval.

### Endovascular intervention

Endovascular stenting of iliocaval occlusion is an attractive option in patients with severe PTS symptoms. This is an area of active research, but high-quality prospective evidence with long duration of follow-up is still lacking. In a 2015 meta-analysis [97] of retrospective and cohort studies, stent placement in those with chronic PTS and iliofemoral venous outflow obstruction (*n* = 1118) resulted in complete pain relief in 69.3% and 1-year primary patency rates of 79%. The major complication is stent thrombosis which has been reported in 13.7% within the initial 6 months, despite concurrent anticoagulation [98]. Re-intervention rates in instances of stent re-thrombosis or occlusion are high and have been reported to be between 15 and 40% within 4 years of stent placement [99]. This is of particular concern in younger patients who require lifelong follow-up and in whom the ramifications to QOL will be significantly impactful. More rigorous and longer-term patency and safety data of this promising intervention is required.

In those with combined superficial venous disease, treatment of superficial venous insufficiency is beneficial. Unfortunately, most patients with PTS will have deep venous valve incompetence and surgical options are limited in this setting. Deep venous valve reconstructions have low rates of long-term success in PTS patients [100]. Bioprosthetic venous valve implants may show promise and there is an ongoing prospective multi-centre study of the porcine VenoValve system (NCT04943172) [101].

## Future directions in prevention of PTS

Patient selection appears to be useful for the targeting of preventative strategies and is likely to be key to ensuring maximum therapeutic benefit. Hence, the development of more effective diagnostic tools such as biomarkers and imaging techniques is important.

### Predictive risk scores

Predictive scoring symptoms may help identify patients who are more likely to develop PTS as we move into the era of personalised medicine. Table 5 summarises the published predictive scoring schemes and their c-statistic scores. There are significant differences in the derivation cohorts of these models, however, which hamper their generalisability.

**Table 5:** Clinical predictive scoring schemes

Model name	Variable	Score	Model performance
SOX-PTS^a^ [42]	Iliac DVT	1	c-stat 0.65
	BMI ≥ 35	2	
	Baseline Villalta score at baseline	10–14 = 1 > 14 = 2	
Mean model^b^ [134]	Age ≥ 75yo	1	c-stat 0.79
	NSAID/antiplatelet	1	
	Multi-level thrombosis	1	
	Prior varicose vein surgery	1	
	Number of leg signs and symptoms	1 for each	
Amin model^c^ [125]	Age > 56	2	c-stat 0.71
	BMI > 30	2	
	Varicose veins	4	
	Smoking	1	
	Provoked DVT	1	
	Iliofemoral thrombosis	1	
	History of DVT	1	
Huang model^d^ [135]	Iliac vein compression	Severe = 2.5 Occlusio* n* = 5.5	Accuracy 81.7%
	Residual iliac–femoral vein thrombosis	3	
	Residual femoral–popliteal vein thrombosis	3	
	Insufficient anticoagulation	6	

^a^After first proximal DVT; ^b^Proximal or distal DVT and > 65 years; ^c^Proximal or distal DVT; ^d^Chinese cohort, first proximal or distal DVT

PTS, post-thrombotic syndrome; DVT, deep vein thrombosis; BMI, body mass index; NSAID, non-steroidal anti-inflammatory drug

### Imaging

A meta-analysis [102] of studies in DVT that correlated CUS findings with PTS found that residual vein thrombus detected between 6 weeks and 12 months is associated with an increased risk of PTS (OR 2.2 95%CI 1.8–2.6). PTS is also predicted by the presence of venous reflux (OR 1.3, 95% CI 1.03–1.7). However, there is significant methodological heterogeneity between studies, particularly in terms of ultrasound timing, CUS techniques and PTS scores. CUS may also be difficult to perform and interpret in patients who have obesity, oedema, unhealed ulcers, or pelvic or inguinal occlusions further hampering diagnostic sensitivity. A recent time-resolved MR venography study found that it could detect veno-lymphatic pathology in one-quarter of CUS negative cases [103]. Future research should focus on determining the best timing and nature of vasculature changes to best predict PTS. There may also be a role for more sensitive modalities like MR venography to fully characterise the changes in veins in patients at high risk of PTS.

### Biomarkers

Identification of a suitable biomarker of PTS may augment diagnosis and improve clinical risk evaluation allowing for better targeting of preventative therapy. Several candidate biomarkers have been explored, the results of which are outlined in this next section.

#### Inflammatory markers and adhesion molecules

Several studies have found C-reactive protein (CRP) to be associated with development of PTS, during the subacute phase between 1 and 12 months after DVT [104]. Other cytokines including IL-6, IL-8, IL-10, TNF-a, RANTES, MCP-1 have shown inconsistent results [104, 105]. Adhesion molecules such as VCAM-1, ICAM-1, P-selectin and E-selectin are expressed by the activated endothelium and are required for leucocyte migration [106]. Of these, ICAM-1 shows the most consistent association with PTS [105, 107, 108], including in the BioSOX study [109] of 703 patients after a first proximal DVT.

#### Matrix metalloproteinases

Matrix metalloproteinases (MMP) are a group of proteolytic enzymes involved in the remodelling of the extracellular matrix and are regulated by tissue inhibitors of metalloproteinases (TIMPs). These play an important part of fibrosis formation post-VTE, which is a major contributor to PTS. In a study of 201 DVT patients [110], MMP-1 and MMP-8 were significantly higher at all time points up to 18 months in PTS patients compared to those without PTS, and TIMP-1 and TIMP-2 were found to be significantly lower at all time points.

#### Marker of thrombosis and fibrinolysis

Most studies have not found differences in levels of factor VIII, von Willebrand factor, fibrinogen, PAI-1, soluble thrombomodulin and peak thrombin in PTS patients compared to non-PTS patients [40, 111–116]. A systematic review [26] found only four studies showing a significant association between elevated D-dimer and PTS, two of which were conducted prior to commencing anticoagulation. Some have shown increased levels of tPA [113] and TAFI [112] in patients who developed PTS.

Global markers of fibrinolytic potential may be a more accurate reflection of overall fibrinolysis. We recently published pilot findings [116] from 190 DVT patients, 32.6% of whom developed PTS (median follow-up 643.5 days). Plasma was sampled from patients during anticoagulation (median 90 days after DVT diagnosis) and overall fibrin generation and fibrinolytic potential measured by the OHP assay. Despite being anticoagulated, patients who subsequently developed PTS showed significantly higher OCP, OHP (indicating increased fibrin generation potential) and reduced OFP% (indication reduced fibrinolytic potential) than those who did not develop PTS. Independent variables associated with PTS are displayed in Table 6. We found two biomarkers to be independent predictors of PTS: OHP and neutrophil/lymphocyte ratio (NLR). These results were incorporated into a predictive multivariate model with a good performance C-stat of 0.77.

**Table 6:** Multivariate logistic model for prediction of developing PTS

	Odds Ratio	Standard Error	p-Value	95% Confidence Interval of Odds Ratio
Proximal DVT	2.22	0.79	0.026	1.10–4.47
History of varicose veins	7.51	4.28	< 0.001	2.46–22.96
NLR ≥ 2.6	2.35	0.95	0.035	1.06–5.18
OH* p* > 13.0 units	2.17	0.79	0.033	1.06–4.43
Weight > 108 kg	2.86	1.11	0.007	1.34–6.11

PTS, post-thrombotic syndrome; DVT, deep vein thrombosis; NLR, neutrophil/lymphocyte ratio; OHP, overall haemostatic potential

The results from our study are novel as they utilise samples collected during the initial anticoagulation period, which removes the need to pause anticoagulation. Previous studies in PTS using CLT, a related global fibrinolytic assay, were performed after the cessation of anticoagulation [112] and found CLT to be correlated with Villalta score (r = 0.38), but not an independent predictor of PTS development. To the best of our knowledge, OHP has not been explored as a predictive biomarker for PTS. Our group has previously reported the findings of pilot studies demonstrating the predictive value of OHP in predicting VTE recurrence in anticoagulated patients [117], as well as oxygen requirement in COVID-19 patients from two waves of the COVID-19 pandemic [118, 119]. As a result, OHP may have a promising prognostic value in various thrombo-inflammatory and hypofibrinolytic diseases, such as PTS.

The neutrophil/lymphocyte ratio (NLR) is an emerging predictive biomarker in cardiovascular and inflammatory conditions [120]. While it has not been studied previously in PTS, elevated NLR has been associated with negative outcomes in venous thrombosis. In the large population-based Tromsø Study [121], NLR > 95^th^ percentile was associated with increased risk of mortality following VTE (adjusted HR 2.13, 95%CI 1.26–3.58, *p* = 0.02). In a meta-analysis of 1424 patients with acute PE, NLR was associated with short-term mortality with negative predictive value 96.7% and positive predictive value 24.4%. NLR is also associated with increased risk of portal vein thrombosis in cirrhotic patients (HR 1.46, 95%CI 1.04–2.04, *p* = 0.028) [122]. These findings support the interconnected nature of the coagulation and inflammatory systems, and our identification of NLR as a predictor of PTS is consistent with this being the outcome of the thrombo-inflammatory state after DVT.

While our study is limited by relatively small numbers and heterogeneous inclusion criteria comprising proximal and distal DVTs, and those with multiple prior DVTs, we have shown that the addition of global coagulation biomarkers has the potential to improve identification of patients at higher risk of developing PTS. The use of these biomarkers should be considered in future randomised clinical studies to help identify the highest risk patients who are likely to benefit from more aggressive preventative interventions.

## Conclusion

Despite recent advances in knowledge, PTS remains one of the most common, chronic and serious complications of DVT, with few effective treatment options. Identifying those who are at high risk of developing PTS, particularly the severe forms, is one of the most difficult challenges. This is the population that may benefit from more intensive preventative measures such as ECS, anticoagulation and invasive treatments. More research is also needed to determine the best target group for catheter-based interventions particularly from a QOL and cost–benefit perspective. Novel biomarkers may play a role in improving existing clinical predictive models, allowing for a more personalised approach.

### Supplementary Information

Below is the link to the electronic supplementary material.

Supplementary file1 (DOCX 23 KB)
