# Supplementary material for: Current challenges in the prevention and management of post-thrombotic syndrome—towards improved prevention
Source: Int J Hematol. 2023 Aug 31;118(5):547–67. doi: 10.1007/s12185-023-03651-6 (PMC10615940; doi:10.1007/s12185-023-03651-6)
Supplement: Supplementary file 1 — Supplementary file1 (DOCX 23 KB) [file 12185_2023_3651_MOESM1_ESM.docx]

**Supplementary materials**

**Medline search strategy**

Keywords used to search article titles and abstracts were:

1. post thrombotic syndrome or post-thrombotic syndrome or postthrombotic syndrome or postphlebitic syndrome
2. treatment or prevention or therapy or intervention or management
3. anticoagulation or warfarin or rivaroxaban or apixaban or dabigatran or edoxaban or heparin
4. elastic compression stockings or graduated compression stockings or compression therapy
5. catheter-directed thrombolysis or catheter-based thrombus removal or pharmacodynamic

Search algorithm: 1 AND (2 OR 3 OR 4 OR 5)

Further requirements for inclusion were

- English language
- Original research
- Published in the last 30 years
- Adults
- Lower limb DVT (upper limb DVT excluded)
- For the elastic compression section, only randomised controlled trials (RCTs) of more than 100 subjects were included
- For the catheter-directed thrombolysis group, only RCTs of more than 100 subjects, and with follow-up more than 6 months were included.

**Table S1.** Villalta score.

| **Symptoms and signs** | **None** | **Mild** | **Moderate** | **Severe** |
| --- | --- | --- | --- | --- |
| ***Symptoms*** |  |  |  |  |
| Pain | 0 | 1 | 2 | 3 |
| Cramps | 0 | 1 | 2 | 3 |
| Heaviness | 0 | 1 | 2 | 3 |
| Paraesthesia | 0 | 1 | 2 | 3 |
| Pruritus | 0 | 1 | 2 | 3 |
| ***Clinical signs*** |  |  |  |  |
| Pretibial oedema | 0 | 1 | 2 | 3 |
| Skin induration | 0 | 1 | 2 | 3 |
| Hyperpigmentation | 0 | 1 | 2 | 3 |
| Redness | 0 | 1 | 2 | 3 |
| Venous ectasia | 0 | 1 | 2 | 3 |
| Pain on calf compression | 0 | 1 | 2 | 3 |
| Venous ulceration | Absent |  |  | Present |

Mild PTS: 5-9; Moderate PTS: 10-14; Severe PTS: ≥15; presence of venous ulcer = score 15.

Abbreviations: PTS, post-thrombotic syndrome;

**Table S2. Ginsberg measure**

| Diagnosis of post-thrombotic syndrome requires the presence of all the following: |
| --- |
| Pain and swelling made worse by standing/walking and relieved by rest/elevation of the leg |
| Duration of symptoms >1 month |
| Diagnosis can be made >6 months after deep vein thrombosis |
| Venous reflux demonstrated by doppler ultrasound or plethysmography |

**Table S3. CEAP classification for diagnosis and grading CVD**

| **Clinical signs** | **Etiology** | **Anatomy** | **Pathophysiology** |
| --- | --- | --- | --- |
| C0 – no visible or palpable signs | Congenital | Superficial | Reflux |
| C1 – telangiectasiae or reticular veins (diameter <3mm) | Primary | Deep | Obstruction |
| C2 – varicose veins (diameter >3mm) | Secondary | Perforator veins | Reflux and obstruction |
| C3 – oedema |  |  |  |
| C4 – changes in skin and subcutaneous tissue secondary to CVD |  |  |  |
| C5 – healed venous ulcer |  |  |  |
| C6 – active venous ulcer |  |  |  |

Abbreviations: CVD, chronic venous disease.
